# Factors associated with post-relapse survival in patients with recurrent cervical cancer: the value of the inflammation-based Glasgow Prognostic Score

**DOI:** 10.1007/s00404-018-4993-0

**Published:** 2018-12-10

**Authors:** Veronika Seebacher, Alina Sturdza, Birgit Bergmeister, Stephan Polterauer, Christoph Grimm, Alexander Reinthaller, Ziad Hilal, Stefanie Aust

**Affiliations:** 1 Gynecologic Cancer Unit, Department of Gynecology and Gynecologic Oncology, Comprehensive Cancer Centre, Medical University of Vienna, Vienna, Austria; 2 Gynecologic Cancer Unit, Department of Radiotherapy, Comprehensive Cancer Centre, Medical University of Vienna, Vienna, Austria; 3 Karl Landsteiner Institute for General Gynecology and Experimental Gynecologic Oncology, Vienna, Austria; 4 Department of Obstetrics and Gynecology, Ruhr-University Bochum, Bochum, Germany

**Keywords:** Cervical cancer, Relapse, Recurrence, Glasgow Prognostic Score, Prognosis

## Abstract

**Purpose:**

The aim of the present study was to assess the value of the Glasgow Prognostic Score (GPS) as a prognostic tool for predicting post-relapse survival (PRS) in patients with recurrent cervical cancer.

**Methods:**

We retrospectively evaluated the data of 116 patients with recurrent cervical cancer in whom serologic biomarkers had been assessed at the time of relapse. The GPS was calculated as follows: patients with elevated serum C-reactive protein levels and hypoalbuminemia were allocated a score of 2, and those with 1 or no abnormal value were allocated a score of 1 and 0, respectively. To assess the association between factors including the GPS and PRS, we performed uni- and multivariate survival analyzes.

**Results:**

After a median follow-up of 20.9 months from recurrence, a 5-year PRS rate of 25% (SE 4.7%) was observed. Only in 29.8% of the patients, recurrence was limited to the pelvis. In uni- and multivariate survival analyzes, the GPS [HR 1.6 (95% CI 0.9–2.4), *p = *0.01], a history of radiation therapy as part of initial treatment [HR 2.7 (95% CI 1.1–6.9), *p =* 0.03], and the presence of peritoneal carcinomatosis or multiple sites of relapse [HR 4.2 (95% CI 1.9–9.3), *p < *0.001] were associated with shorter PRS. The GPS correlated with higher squamous cell carcinoma antigen levels (*p* = 0.001), shorter median PRS (*p* = 0.009), and less intensive treatment for relapse (*p* = 0.02).

**Conclusions:**

A higher GPS at the time of relapse, a history of radiation therapy, and the presence of peritoneal carcinomatosis or multiple sites of relapse are independently associated with shorter PRS in patients with recurrent cervical cancer.

## Introduction

Since the provision of screening programs, the incidence and mortality of cervical cancer in developed countries have decreased by 75% [1]. Unfortunately, in countries without access to screening and prevention programs, cervical cancer still remains the second most common type of cancer in women. Depending on initial tumor stage, between 8 and 61% of women with cervical cancer experience relapse of disease, most commonly within the first 2 years of completing the treatment [2]. Treatment in these patients is individualized according to the site of relapse and the patient’s performance status. In only a few patients, long-term survival can be achieved by radical surgery or radiotherapy of a central pelvic relapse or solitary distant metastasis [3]. Other than that, platinum-based chemotherapy is the mainstay for these patients. Compared to chemotherapy alone, by adding the angiogenesis inhibitor bevacizumab, response and survival rates can be improved by several months [4]. However, the majority of patients will suffer further disease progression and death within a median time of 11–16 months [5–7]. Few studies have addressed the question of prognostic parameters for post-relapse survival (PRS). A small series of 45 patients with advanced stage at presentation, short disease-free interval (DFI), and recurrence within the previously irradiated field were found to be independently associated with shorter PRS [5]. Another study in 121 patients reported extravaginal site of relapse and HPV 16-positive cancer to be independent prognostic factors [6]. Furthermore, secondary radical surgery for relapse treatment was reported as the only independent prognostic factor for PRS in a series of 74 patients with recurrent cervical cancer [7]. The largest study prospectively evaluated pooled clinical prognostic factors known as the Moore criteria in 452 patients treated within the Gynecologic Oncology Group protocol 240 study [8]. Including black race, performance status 1, pelvic disease, prior cisplatin, and DFI < 365 days, high risk according to the Moore criteria were associated with worse overall survival and better response to bevacizumab-containing treatment. Yet, the authors suggested that additional models including factors such as nutritional status may have further clinical utility.

Chronic inflammation is known as the hallmark of carcinogenesis and is substantially involved in the multistage process of tumor metastasis [9]. As the driving force behind metabolic alterations, inflammation is a key factor in the development of muscle wasting, a central component of cancer cachexia [10]. The inflammation-based Glasgow Prognostic Score (GPS) is a scoring system combining elevated C-reactive protein (CRP) serum levels and hypoalbuminemia, factors for inflammation and hypermetabolism, used to stratify patients into risk groups for predicting clinical outcome. Among others, our study group has previously evaluated the role of the GPS in cervical cancer patients, and found higher GPS, assessed prior to initial treatment, to be independently associated with shorter survival [11, 12].

The aim of the present study was to investigate the prognostic role of several clinical parameters and the GPS, assessed at the time of diagnosis of first relapse, for PRS in patients with recurrent cervical cancer.

## Materials and methods

### Patients’ cohort and data acquisition

We included all patients who were treated for cervical cancer at the Medical University of Vienna between 1998 and 2014, and who experienced recurrent disease after response to initial treatment. Patients who had disease progression during initial treatment were excluded from analysis. Patients’ records were reviewed to identify patients with serological measurements of albumin and CRP available at the time of diagnosis of first relapse of cervical cancer. Only these patients were included in the present study.

The study was approved by the institutional review board, i.e., the ethics committee of the Medical University of Vienna (Project 2160/2016). Due to the retrospective design of the study, the ethics committee did not require informed consents of the study participants. Patient’s data were de-identified and handled in accordance with ethical standards of good scientific practice.

### Clinical management

Initial management of patients with cervical cancer was adjusted according to the International Federation of Gynecology and Obstetrics (FIGO) tumor stage. Microinvasive cervical cancer (FIGO Ia1) was treated either with conization or simple hysterectomy, in case of lymphovascular space involvement (LVSI) with an additional pelvic lymphadenectomy. Early-stage disease (FIGO Ia2–IIa) was managed with radical hysterectomy and pelvic lymphadenectomy. In case of lymph node metastases, hysterectomy was omitted, periaortic lymphadenectomy was performed, and patients were treated by primary chemoradiation therapy. Patients with intermediate risk according to Sedlis criteria [13] received adjuvant (chemo-)radiation therapy. Locally advanced cervical cancer or early-stage cervical cancer with lymph node metastases was treated by concurrent chemoradiation therapy including image-guided brachytherapy (IGBT). Of note, prior to 2001, patients were treated by primary radiation therapy without concurrent chemotherapy. To assess lymph node status, patients had pelvic and/or periaortic lymph nodes removed prior to radiation therapy. Metastatic cervical cancer (FIGO IVb) was treated by individually tailored palliative radiation and chemotherapy, as recommended in the multidisciplinary tumor board.

Following initial treatment, patients were included in our institution’s standardized gynecologic oncology follow-up program. For the first 3 years, patients were followed up every 3 months, in the fourth and fifth year biannually, and yearly from the sixth to the tenth year after primary treatment. Computed tomography (CT), and in selected cases magnetic resonance imaging (MRI) or positron emission tomography (PET)–CT, was performed on a yearly basis. Furthermore, if clinically suggested or in case of elevation of tumor markers, imaging was performed as indicated. Recurrent disease was either diagnosed clinically, by biopsy or imaging methods. Death and its cause were documented based on the autopsy results and the records in death certificates.

### Albumin and CRP measurement

Blood samples (serum) were obtained at time of diagnosis of first relapse of cervical cancer, before initiation of any treatment. Assessment of serum albumin and CRP was done at the central laboratory of our institution. Serum albumin was assayed with bromocresol green using routine clinical chemical photometric analyzers [14] (Hill 1985). For the measurement of serum CRP levels, a commercially available immunoturbidimetric test (Olympus, CRP Latex; Olympus Life and Material Science Europe, Hamburg, Germany) was used.

### Glasgow Prognostic Score

The GPS was calculated as previously described [11]: patients with elevated serum CRP levels (> 10 mg/l) and hypoalbuminemia (< 35 g/l) were allocated a score of 2, and patients with 1 or no abnormal value were allocated a score of 1 or 0, respectively.

### Statistical analysis

Categorical variables are presented as numbers and proportions, and continuous variables as medians [interquartile range (IQR)]. Group differences in categorical and continuous variables were analyzed using chi-square and Kruskal–Wallis tests, respectively. DFI was calculated from the date of initial diagnosis to the date of first relapse. For survival analyzes, we assigned patients to risk groups according to the GPS (2 vs. 1 vs. 0), serum SCC–Ag (> vs. < 1.5 ng/ml), treatment of relapse (surgery vs. no surgery), the site of relapse (peritoneal carcinomatosis or multiple sites vs. others), DFI (< 6 vs. 6–12 vs. 13–24 vs.> 24 months), radiation therapy at initial treatment (yes vs. no), BMI (< vs. ≥ 25 kg/m^2^), histologic subtype (SCC vs. non-SCC), FIGO tumor stages (IV vs. III vs. II vs. I), and age groups at time of relapse (< 40 vs. 40–49 vs. 50–59 vs.≥ 60 years). Survival probabilities for each risk group were calculated by the product limit method of Kaplan and Meier. Differences between groups were tested using the log-rank test. The results were analyzed for the endpoint of PRS. Post-relapse survival (PRS) was calculated from the date of first relapse to the date of death for all cause or the date of the last follow-up. For PRS, events were defined as cervical cancer-related death and death due to any cause. Patients who were still alive were censored with the date of last follow-up. Multivariate Cox regression models were performed including all risk groups associated with PRS in univariate analyzes. Results of uni- and multivariate survival analyzes are given as p value (hazard ratio (HR) and 95% confidence interval (95% CI)]. *p* values < 0.05 were considered statistically significant. We used the statistical software SPSS 16.0 for Mac (SPSS 24.0.0, SPSS Inc., Chicago, IL, USA) for statistical analyzes.

## Results

Of the 685 patients treated for cervical cancer at our institution between 1998 and 2014, relapse of disease after initial response to treatment occurred in 145 (21.1%) patients. Serological measurements of CRP and albumin at the time of diagnosis of first relapse were available in 116 patients, who were, therefore, eligible for the present study.

At the time of initial diagnosis, the patients’ median age (IQR) was 50.6 years (40.8–60.7). Cervical cancer was diagnosed at tumor stages FIGO Ia1 in 2 (1.7%), FIGO Ib1 in 15 (12.9%), FIGO Ib2 in 8 (6.9%), FIGO IIa in 12 (10.3%), FIGO IIb in 50 (43.1%), FIGO IIIa in 1 (0.9%), FIGO IIIb in 19 (16.4%), FIGO IVa in 8 (6.9%), and FIGO IVb in 1 (0.9%). In 92 patients (79.3%), squamous cell carcinoma, in 20 (17.2%), adenocarcinoma, and in 4 (3.5%), adenosquamous carcinoma were found. Twenty-one patients (18.1%) underwent radical hysterectomy with pelvic lymphadenectomy. Of these, four patients (3.5%) received neoadjuvant chemotherapy and seven (6%) adjuvant radiotherapy. In one patient each (0.9%), a radical trachelectomy with pelvic lymphadenectomy, a simple hysterectomy, and a cone biopsy were done as initial treatment. Eleven (9.4%) and 81 patients (69.8%) were initially treated with primary radiation therapy and primary chemoradiation therapy, respectively. In 75 patients (64.6%), regional lymph nodes were removed prior to radiation therapy with pelvic and periaortic lymphadenectomy in 71 (61.2%) and in 21 patients (18.1%), respectively. In 27 patients (23.3%), external beam radiation was extended to the periaortic region. Following external beam radiation, 92 (79.3%) patients underwent IGBT. One hundred five patients (90.5%) had complete and 11 (9.5%) partial response to primary treatment.

Clinical and pathological characteristics at the time of first relapse of cervical cancer for all the patients and stratified into GPS risk groups are given in Table 1. There was no difference in the distribution of median age or BMI at time of relapse, DFI from primary diagnosis, and site of relapse between GPS risk groups. Of note, more patients with GPS 1 and 2 had elevated serum SCC–Ag levels compared to those with GPS 0 (*p = *0.001). In the majority of patients (68.9%), distant metastases were found at time of first relapse. Extra-abdominal lymphatic spread included metastases of inguinal, cervical, axillary, and mediastinal lymph nodes. Visceral metastases were found in the lung in 26 (22.4%), the liver in 5 (4.3%), the skin in 4 (3.5%), the spleen in 2 (1.7%), and the brain in 1 patient (0.9%). Compared to patients with GPS 0, fewer patients with GPS 1 and 2 underwent surgery for first relapse, and a higher rate received no treatment (*p = *0.02). In addition, median post-relapse survival was inversely correlated with the GPS (*p = *0.009). Results of the treatment and post-relapse follow-up for all the patients, and stratified into GPS risk groups are shown in Table 2.

**Table 1:** Patients’ characteristics at time of relapse in 116 patients with recurrent cervical cancer, adjusted to Glasgow Prognostic Score risk groups

	Median/*N* (IQR/%)				*p* value
	All	GPS 0	GPS 1	GPS 2	
Number of patients	116 (100)	41 (35.3)	56 (48.3)	19 (16.4)	
Median age at time of relapse, years (IQR)	52.1 (42.3 – 62.6)	51.4 (40.5–60)	53.3 (43.8–65.9)	53.4 (1.6–70.2)	0.4*
Median DFI from primary diagnosis, months	12.7 (8.4–28.1)	17.5 (10.9–34)	11.9 (7.9–21.7)	10.4 (7.8–26.2)	0.1*
DFI from primary diagnosis					0.3^#^
<6 months	10 (8.6)	3 (7.3)	5 (8.9)	2 (10.5)	
6–12 months	45 (38.8)	11 (26.8)	25 (44.7)	9 (47.4)	
13–24 months	28 (24.1)	14 (34.1)	13 (23.2)	1 (5.3)	
>24 months	33 (28.5)	13 (31.8)	13 (23.2)	7 (36.8)	
BMI					0.9^#^
Underweight	13 (11.2)	3 (7.3)	7 (12.5)	3 (15.8)	
Normal weight	49 (42.2)	22 (53.7)	20 (35.7)	7 (36.8)	
Overweight/obese	32 (27.6)	13 (31.7)	13 (23.2)	6 (10.7)	
NA	22 (19)	3 (7.3)	16 (28.6)	3 (15.8)	
SCC–Ag					0.001^#^
≤ 1.5 ng/ml	34 (29.3)	20 (48.8)	10 (17.9)	4 (21.1)	
> 1.5 ng/ml	52 (44.8)	11 (26.8)	34 (60.7)	7 (36.8)	
NA	30 (25.9)	10 (24.4)	12 (21.4)	8 (42.1)	
Site of relapse					0.2^1,#^
Pelvis	36 (31)	13 (31.7)	17 (30.4)	6 (31.6)	
Distant	80 (70)	28 (68.3)	39 (69.6)	13 (68.4)	
Paraaortic lymph node metastasis	15 (12.9)	4 (9.8)	9 (16.1)	2 (10.5)	
Extra-abdominal lymphatic spread	10 (8.6)	4 (9.8)	6 (10.6)	0	
Peritoneal carcinomatosis	6 (5.2)	0	3 (5.4)	3 (15.8)	
Visceral metastasis	38 (32.8)	19 (46.3)	14 (25)	5 (26.3)	
Bone metastasis	4 (3.5)	0	3 (5.4)	1 (5.3)	
Multiple sites	7 (6.0)	1 (2.4)	4 (7.1)	2 (10.5)	

*IQR* interquartile range, *DFI* disease-free interval, *BMI* body mass index (underweight: BMI < 18.5 kg/m^2^; normal weight: BMI 18.5–24.9 kg/m^2^; overweight/obese: BMI ≥ 25 kg/m^2^); *SCC–Ag* squamous cell carcinoma antigen, *NA* not available

^1^Distant vs. pelvic relapse

*Kruskal–Wallis test

^#^Chi-square test

**Table 2:** Treatment and post-relapse follow-up in 116 patients with recurrent cervical cancer, adjusted to Glasgow Prognostic Score risk groups

	Median/*N* (IQR/%)				*p *value
	All	GPS 0	GPS 1	GPS 2	
Number of patients	116 (100)	41 (35.3)	56 (48.3)	19 (16.4)	
Treatment of relapse					0.02^#^
Palliative	10 (8.6)	2 (4.9)	5 (8.9)	3 (15.8)	
Surgery	7 (6.1)	1 (2.4)	5 (8.9)	1 (5.3)	
Surgery plus chemo and/or radiation therapy	16 (13.8)	9 (21.9)	5 (8.9)	2 (10.5)	
Radiation therapy	19 (16.4)	5 (12.2)	8 (14.3)	6 (31.6)	
Chemotherapy	43 (37)	9 (21.9)	29 (51.8)	5 (26.3)	
Chemo- and radiation therapies	21 (18.1)	15 (36.7)	4 (7.2)	2 (10.5)	
Median post-relapse survival, months	10.2 (5.5–21.1)	16.1 (9.2–40.9)	8.7 (4.4–16.7)	6.9 (3.1–12.2)	0.009*
Status at time of last observation					0.5^#^
Free of disease	13 (11.2)	6 (14.6)	6 (10.7)	1 (5.3)	
Stable disease	11 (9.5)	7 (17.1)	3 (5.4)	1 (5.3)	
Progressive disease	17 (14.7)	6 (14.6)	9 (16.1)	2 (10.5)	
Non-cancer-related death	3 (2.6)	1 (2.4)	2 (3.6)	0	
Cancer-related death	72 (62.1)	21 (51.2)	36 (64.3)	15 (78.9)	

*IQR* interquartile range, *GPS* Glasgow Prognostic Score

*Kruskal–Wallis test

^#^Chi-square test

**Fig. 1:**
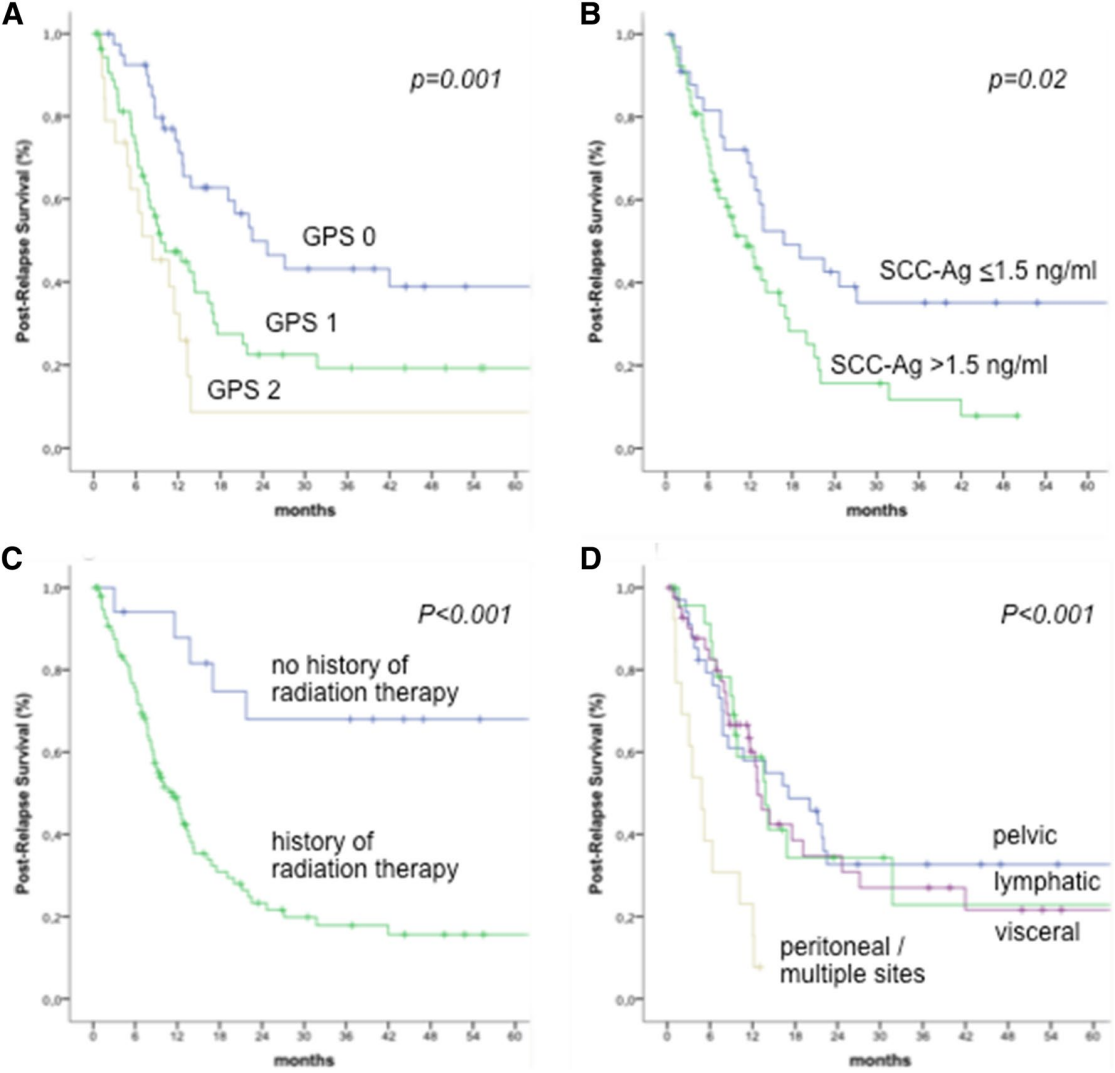
Kaplan–Meier curves for post-relapse survival stratified into the risk groups. **a** Glasgow Prognostic Score, **b** squamous cell carcinoma antigen, **c** history of radiation therapy, and **d** site of relapse

After a median (IQR) follow-up of 20.9 (9.5–51.4) months from recurrence, 72 patients (62.1%) died from progressive disease and 3 patients (2.6%) from other cause, leading to a 5-year PRS rate of 25% (SE 4.7%). Results of uni- and multivariate analyzes for PRS investigating the effect of selected clinical, pathological and serological parameters are shown in Table 3. Among all parameters investigated, a history of radiation therapy as part of initial treatment (*p* < 0.001), the presence of peritoneal carcinomatosis or multiple sites of relapse (*p* < 0.001), elevated serum SCC–Ag (*p* = 0.02), and the GPS (*p* = 0.001) were the only factors associated with shorter PRS. Of these, a history of radiation therapy, the presence of peritoneal carcinomatosis or multiple sites of relapse, and the GPS remained as independent prognostic factors for PRS after adjusting for reciprocal effects in multivariate analysis. Kaplan–Meier curves for PRS stratified into risk groups are shown in Fig. 1.

**Table 3:** Association between clinical, pathological, and serological risk factors and post-relapse survival in 116 patients with recurrent cervical cancer

	Univariate		Multivariate^#^	
	p*	HR (95% CI)^#^	*p*	HR (95% CI)
Age groups at time of relapse^a^	0.2	1.1 (0.9–1.3)	–	–
FIGO stages (IV vs. III vs. II vs. I)	0.2	1.2 (0.9–1.5)	–	–
Histologic subtype (SCC vs. non-SCC)	0.9	1.0 (0.6–1.8)	–	–
BMI (< vs. ≥ 25 kg/m^b^)	0.6	1.1 (0.7–1.9)	–	–
Radiation therapy at initial treatment (yes vs. no)	< 0.001	4.7 (1.9–11.9)	0.03	2.7 (1.1–6.9)
DFI (<6 vs. 6–12 vs. 13–24 vs.>24 months)	0.3	0.8 (0.7–1.1)	–	–
Site of relapse^b^	< 0.001	4.1 (2.1–7.8)	<0.001	4.2 (1.9–9.3)
Treatment of relapse (surgery vs. no surgery)	0.1	1.6 (0.8–3.1)	–	–
SCC–Ag (> vs. ≤ 1.5 ng/ml)	0.02	1.9 (1.1–3.4)	0.1	1.5 (0.9–2.4)
GPS (2 vs. 1. vs. 0)	0.001	1.8 (1.3–2.5)	0.01	1.6 (1.1–2.5)

*HR* hazard ratio, *CI* confidence interval, *SCC–Ag* squamous cell carcinoma antigen, *GPS* Glasgow Prognostic Score

*Kaplan–Meier analysis

^#^Cox regression analyzes

^a^ < 40 years vs. 40–49 years vs. 50–59 years vs. 60+ years; SCC squamous cell carcinoma

^b^Site of relapse: peritoneal carcinomatosis/multiple sites vs. visceral/bone/lymphatic/pelvic relapse

## Discussion

Estimation of the prognosis of PRS in patients with recurrent cervical cancer is essential to tailor treatment to the individual’s need. Hitherto, few studies have addressed the quest for prognostic factors for PRS in these patients. To the best of our knowledge, this is the first study to investigate the usefulness of serological parameters for inflammation and hypermetabolism as prognostic factors in the setting of disease recurrence in patients with cervical cancer. Our results demonstrate the value of the GPS for predicting PRS in patients with recurrent cervical cancer, independent of other factors associated with PRS.

Both chronic inflammation and nutritional decline are substantial characteristics of cachexia in cancer patients. As a multifactorial, multi-organ syndrome involving changes in several metabolic pathways, cachexia is indirectly responsible for the death of approximately 20% of all the cancer patients [10, 15]. Cytokines, released by activated immune cells and tumor cells, promote the activation of transcription factors associated with wasting of skeletal muscle and adipose tissue [10]. As a consequence, an important flow of amino acids from skeletal muscle to the liver is found in cancer patients, serving for both gluconeogenesis and acute-phase protein synthesis. Veritably, elevated CRP, one of the major acute-phase proteins synthesized by the liver, was found to be associated with advanced disease and shorter survival in various malignancies, including cervical cancer [16–18]. Another biomarker associated with cachexia and impaired prognosis in cancer patients is hypoalbuminemia [19–21]. A complex interplay between changes in albumin degradation rates, increased capillary permeability, and decreased hepatic synthesis seems to be responsible for low serum albumin levels in cancer patients [22, 23].

Based on the combination of serum CRP and serum albumin, the GPS is a well-established, cheap and readily available tool for prognostic assessment in cancer patients [20, 24, 25]. In cervical cancer, pre-treatment GPS was reported to be associated with more advanced disease, higher tumor grade, and lymph node involvement. Furthermore, a higher GPS was predictive of shorter overall survival, independent of tumor stage and lymph node involvement [11, 12]. In accordance with these studies, our results demonstrate a strong relationship between higher GPS at the time of diagnosis of first relapse and shorter PRS in patients with recurrent cervical cancer. This association remained unchanged even after adjusting the effects of other clinical parameters associated with PRS in multivariate analysis.

In addition to the GPS, we evaluated the prognostic value of several clinical and pathological factors. The only factors we found to be associated with shorter PRS were a history of radiation therapy as part of initial treatment, the presence of peritoneal carcinomatosis or multiple sites of relapse, and elevated serum SCC–Ag levels. Yet, in multivariate analysis only the GPS, a history of radiation therapy, and the site of relapse remained independently associated with shorter PRS.

We did not find any other study reporting on a negative effect of a history of radiation therapy as part of initial treatment for PRS in patients with cervical cancer. As we included patients of all tumor stages, a history of radiation can be regarded as a surrogate parameter for advanced disease or tumors with high-risk features at the time of initial presentation. Nevertheless, FIGO tumor stage at initial presentation was not associated with PRS in our cohort. Moreover, recurrence within a previously irradiated field was reported to be less responsive to salvage chemotherapy [5]. Radiation causes vasculitis that limits adequate drug distribution and perfusion into the irradiated tumor beds. Furthermore, the majority of patients have received platinum-containing chemotherapy concomitant to radiation therapy causing platinum resistance [8].

Another interesting finding of our study was the association between the site of relapse and PRS. Of note, only the presence of peritoneal carcinomatosis or multiple sites of relapse were associated with shorter PRS. There was no difference in PRS between pelvic, lymphatic and visceral sites of relapse. This finding is in contradiction to a Taiwanese study in 121 patients with recurrent cervical cancer that found extravaginal site of relapse to be independently associated with shorter PRS [6]. However, while in this study all patients had radical hysterectomy as primary treatment, and only 45% received adjuvant radiation therapy, nearly 86% of the patients in our cohort were initially treated by either primary radiation therapy or adjuvant radiation therapy following radical hysterectomy. Other than that, our results were in accordance with an Italian study in 75 patients with recurrent locally advanced cervical cancer after primary multimodality therapy [7]. Likewise, this study found PRS to be shortest in those patients with peritoneal and mixed recurrences, followed by those with pelvic site of relapse. Those with distant metastases had longest PRS. Hence, this would strengthen our finding on the negative effect of radiation therapy on PRS, suggesting that salvage treatment might not be as effective in previously irradiated areas.

By comparing the distribution of patients’ characteristics between the respective GPS risk groups we found elevated serum SCC–Ag levels to be associated with GPS risk groups 1 and 2. As serum SCC–Ag is a well-known marker for tumor stage and size in cervical cancer, this correlation might suggest the GPS to be a surrogate for higher tumor burden [26].

In addition, within GPS risk groups 1 and 2 more patients had palliative treatment only, and fewer patients had surgical treatment for relapse compared to patients with a GPS 0. Again, this reiterates that a higher GPS reflects advanced tumor burden, and, eventually, less favorable performance status. Type of treatment for relapse was not associated with PRS. Therefore, we do not assume the prognostic value of the GPS for PRS to be only a surrogate marker for less treatment.

Strengths of the present study include the standardized follow-up program for all patients at our institution. Therefore, only very few recurrences could have been missed. Within the follow-up program, patients had CT or PET–CT scans done on a regular, annual basis. This facilitates the ascertainment of the time point of first relapse and the pattern of relapse. However, there are some limitations that deserve to be mentioned. Within this study, data were retrospectively analyzed, leading to shortcomings, such as patient selection and incomplete data acquisition. In addition, due to the relatively long study period, type of imaging to detect relapse and type of treatment might have changed over time.

In conclusion, the present study suggests the GPS to independently predict PRS in patients with recurrent cervical cancer. In addition, a history of radiation therapy as initial treatment and the presence of peritoneal carcinomatosis or multiple sites of relapse were associated with shorter PRS. Our results contribute to the relatively small amount of literature on factors associated with PRS in recurrent cervical cancer and might be helpful in patient counseling. Yet, larger studies are required to validate our results.
